# Investigating the Use of Smartphones for Learning Purposes by Australian Dental Students

**DOI:** 10.2196/mhealth.3120

**Published:** 2014-04-30

**Authors:** Andrea Rung, Frauke Warnke, Nikos Mattheos

**Affiliations:** ^1^Griffith UniversitySchool of DentistryGold CoastAustralia; ^2^The University of Hong KongFaculty of DentistryHong Kong

**Keywords:** health care education, smartphone, mobile technology, social media, computer literacy

## Abstract

**Background:**

Mobile Internet devices and smartphones have at present a significant potential as learning tools and the development of educational interventions based on smartphones have attracted increasing attention.

**Objective:**

The objective of this study was to obtain a deeper insight in the nature of students’ use of smartphones, as well as their attitudes towards educational use of mobile devices in order to design successful teaching interventions.

**Method:**

A questionnaire was designed, aiming to investigate the actual daily habitual use, as well as the attitudes of dental students towards smartphones for their university education purposes. The survey was used to collect data from 232 dental students.

**Results:**

Of the 232 respondents, 204 (87.9%) owned a smartphone, and 191 (82.3%) had access to third generation (3G) mobile carriers. The most popular devices were the iPhone and Android. Most of the respondents had intermediate smartphone skills and used smartphones for a number of learning activities. Only 75/232 (32.3%) had specific educational applications installed, while 148/232 (63.7%) used smartphones to access to social media and found it valuable for their education (*P*<.05). Students accessing social media with their smartphones also showed significantly more advanced skills with smartphones than those who did not (*P*<.05). There was no significant association between age group, gender, origin, and smartphone skills. There was positive correlation between smartphone skills and students' attitudes toward improving access to learning material (*r*=.43, *P*<.05), helping to learn more independently (*r*=.44, *P*<.05), and use of smartphones by teaching staff (*r*=.45, *P*<.05).

**Conclusion:**

The results in this study suggest that students use smartphones and social media for their education even though this technology has not been formally included in the curriculum. This might present an opportunity for educators to design educational methods, activities, and material that are suitable for smartphones and allow students to use this technology, thereby accommodating students’ current diverse learning approaches.

## Introduction

Educational methods must be dynamic and continuously adapt to an ever-changing social environment [1]. Information and communication technology (ICT) has been a critical component of teaching and learning in higher education over the last few decades. One particularly important trend we have recently witnessed with regard to the use of ICT is the increasing reliance on mobile-connected devices not only in daily tasks, but also within professional and educational environments [2].

Without a doubt, the effective use of mobile devices today has become one significant parameter of “computer literacy.” Consequently, primary and high schools are increasingly introducing mobile technology to enhance teaching and learning. It is not surprising, therefore, that students expect to use this technology when attending university courses [3,4].

Evidence of the current widespread use of smartphones in medical education has been reported in a Canadian study where not only 85% of medical students and faculty used smartphones daily, but they also expected the usage of this technology to increase in medical education and practice [5]. While medical students in the United Kingdom also reported to expect the usage of smartphones to be beneficial and likely to increase in the future, a reduced number of students reported owning and using smartphones [6].

The current generation of health care students has grown up surrounded by information technology. “Millennials,” or those born after 1982, have embraced mobile technology and social media. It has been reported that social media can improve participation and link diverse and geographically dispersed groups of students and professionals [7] by enabling communication outside the classroom, improving collaboration, creativity, and connecting students with experts [8].

The use of mobile technology can significantly enhance blended learning [9], but can have a major role in also supporting on-campus teaching. Smartphones have been used in educational activities to access course content, acquire information related to students’ performance, and to encourage discussion and sharing between students and teachers [10]. It is therefore apparent that mobile devices such smartphones can have a significant contribution to modern health care education, since these devices might offer possibilities to enhance teaching and learning.

As with every technology, however, understanding the skills of the main users and their attitudes toward the new tool is of fundamental importance, in order to guide development of appropriate educational innovation. At times, students have been reported to be reluctant to use smartphones for learning; they would rather use their smartphones for social and private activities [3].

Few reports are presently available on educational innovations with the use of smartphones. While they are often testing the use of specific applications or programs operating in smartphones [11-22], little is known on how students perceive their smartphones as an educational tool at their own initiatives and outside the framework of specific applications.

The purpose of this study was to objectively investigate whether and up to what extent dental students use their smartphones as learning tools in the first 3 years of their dental education, in the absence of a specific application or requirement provided by the university. In addition, the subjective attitudes of students toward smartphones as learning tools were to be investigated.

## Methods

### Ethics Approval

This study was approved by the Research Ethics Committee of Griffith University, Gold Coast, Australia.

### Questionnaire

A descriptive questionnaire survey was developed, aiming to assess not only students’ subjective attitudes, but also to provide an objective understanding of the extent and complexity in which students have used smartphones. The questionnaire was tested for face validity with a group of undergraduate students (Multimedia Appendix 1). For the purpose of this study, mobile use “for learning purposes” was extended to include any use that facilitates or relates to the learning process and educational activities. In that sense, looking at the timetable or course announcements was included, although such use does not constitute a direct learning activity, it does however facilitate the learning process and is part of the day to day educational process. The questionnaire was structured in three parts: (A) demographics, (B) assessment of use, and (C) assessment of attitudes.

The first part, part A, included demographic and social characteristics, as well as the type of smartphone and connection used by students.

Part B was composed of questions, which explored the nature and complexity of the tasks carried out by students with their smartphones, and in particular its use for learning purposes, including the use of social media. As the use of a smartphone is today perceived as an important parameter of computer literacy, the questionnaire was modelled after a widely-used design aimed to objectively assess computer literacy [23,24]. In that model, students were called to respond whether they had or had not performed a series of tasks of increasing complexity. These tasks included communication, access and sharing of information, commercial transactions, and creation of content such taking pictures and making movies. An area was available for students to add other tasks they might perform with the smartphone.

As based on the previous model [23,24], the sum of every positive response gave a score with the maximum possible score of 16. On the basis of the tasks, the ranges of scores 0-5 were categorized as basic, 6-10 as intermediate, and 11-16 as advanced.

Part C, the third part, aimed to measure some basic subjective attitudes toward the use of smartphones as educational tools. This was done by stating the degree of agreement/disagreement with three statements on a visual analogue scale (VAS).

### Sample

The questionnaire was distributed to first- (n=126), second- (n=117), and third- (n=78) year dental students. At the time of this study, the curriculum used a learning management system through which digital content was made available at several occasions. However, the curriculum did not include any methodology, content, or application that involved the specific use of a mobile device.

### Analysis

Data were analyzed with descriptive statistics. Chi-square tests were used to calculate the correlations between demographic elements and scores, while linear regression tests were used to calculate correlations between demographics and attitudes. The analysis was done with SPSS version 21. Absolute values were used with percentages to indicate unanswered questions. Correlations were tested at 95% significance level (*P*<.05).

## Results

### Demographic Characteristics

In total, 72.2% (232/321) of students returned the questionnaires. One student did not fill in the demographic information, although he filled in all remaining parts. As a result the demographic data was received from 231 students. Of these 231 students, 193 (83.5%) were domestic (Australians) and 38 (16.5%) were from overseas. There were 130 male (56.2%) and 101 female (43.7%) respondents. One hundred and six students (106/231, 45.7%) reported having a part-time job.

### Type of Smartphone and Connection

Smatphones were owned by 204/223 (91.5%) students, and 191/214 (89.2%) of the respondents had access to Internet data through a third generation (3G) mobile carrier. The devices used by students were as follows: iPhone (111/213, 52.1%), Androids (96/213, 45.0 %), Windows (4/213, 1.9%), and Blackberry (2/213, 0.1%).

Only 42/214 (19.6%) students owned a tablet, while 48/214 (22.4%) might be buying a tablet in the next few months, and 124/214 (57.9%) were unlikely to buy a tablet in the near future.

### Assessment of Use

**Figure 1:**
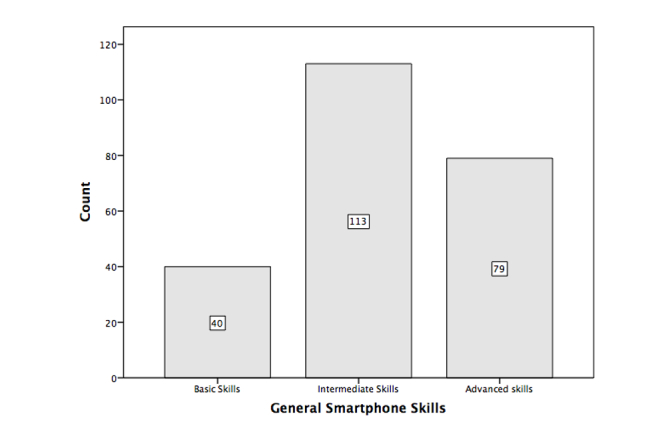
Number of students with basic (n=40), intermediate (n=113), and advanced skills (n=79).

The average skills score with smartphones (as described in part B) was 8.52. This score was categorized as corresponding to intermediate skills. There were no significant differences in skills between age groups or gender (Figure 1, Table 1).

**Table 1:** Smartphones general skills scores per age group reported as mean, SD, and number of students per group.

Age by groups	Mean	SD	n (%)
17 to 20	8.30	3.7	120 (52.6)
21 to 25	8.75	4.6	57 (25.0)
26 to 30	9.12	4.3	26 (10.9)
≥31	8.00	4.3	25 (10.9)
Total	8.47	4.1	228 (100.0)

### Use of Smartphones and Social Media for Learning

The smartphone features that students were more likely to use for learning purposes were: looking at the timetable and course announcements, followed by surfing the Web for learning material, and taking pictures of their work (Table 2). There were not significant differences between age, gender, and international or domestic students’ response.

**Table 2:** Number and percentage of students using their smartphones for learning activities.

Activity	Students	
	n	%
Looking course timetable	177	83.1
Course announcements	175	82.2
Surf the Web for material	139	65.5
Picture of my work	139	65.3
Email staff/classmates	132	62.0
Read lecture notes	118	55.4
Share notes	86	40.4
Library/literature search	63	29.7
Watch instructional movie	52	24.5
Watch lectures	48	22.7
Make movies of my work	19	9.0

Only 76/204 (37.3%) responded to have dental and/or educational applications in their phones. Whenever reported, these included applications with quizzes on anatomy and chemistry. The Griffith University smartphone application [25] was present in 126/214 (58.8%) smartphones and 89/145 (61.3%) students stated that they use it regularly or often. Students were regularly or often using their smartphones on the go (156/208, 75.0%), on campus (154/207, 74.3%), at home (125/208, 60.1%), and in the lecture theater (116/206, 53.6%).

Some respondents found social media valuable for their education (155/201, 77.1%), and a significant number of them (148/201, 73.6%) accessed social media using their smartphones (*P*<.05) (Table 3). Students accessing social media with their smartphones also showed significantly more advanced skills with their smartphones than those who did not (*P*<.05) (Table 3). Age group, gender, or type of smartphone did not show significant association with the smartphone skills.

Students believed that social media enabled them to collaborate by sharing notes and tips, while it also helped them to stay informed. Younger students were more likely to access to social media with their smartphones than older ones (Table 4).

**Table:** Cross tabulation comparing number of students accessing social media with smartphones, and the number of students who find social media valuable for learning, and the number of students who accessed social media with smartphones and students’ skills with the smartphone (chi-square *P*<.05).

		Access to Social Media				Total	
		Yes		No			
		n	%	n	%	n	%
**Value Social Media**							
	Yes	148	95.4	7	4.5	155	77.1
	No	30	65.2	16	34.7	46	22.8
	Total	178	88.5	23	11.4	201	100.0
**Smartphone Skills**							
	Basic	13	56.5	10	43.4	23	10.9
	Intermediate	19	16.9	14	12.5	112	53.3
	Advanced	74	98.6	1	1.3	75	35.7
	Total	185	88.0	25	11.9	210	100.0

**Table 4:** Cross tabulation comparing number and percentage of students who access to social media by age group (Cross tabulation chi-square *P*=.035).

Age group	Yes		No		Total	
	%	n	%	n	%	n
17-20	94.7	104	6.3	7	53.9	111
21-25	86.0	43	14.0	7	24.3	50
26-30	81.0	17	19.0	4	10.2	21
≥31	75.0	18	25.0	6	11.7	24
Total	88.3	182	11.7	24	100.0	206

###  Assessment of Attitudes

The strongest attitude expressed through the VAS was that smartphones help improve access to the courses learning material (mean VAS score 7.21, SD 1.9). A lighter agreement appeared with smartphones enabling students to learn more independently (mean 6.1, SD 2.2), while a slightly stronger agreement was that teaching staff should use smartphones for teaching (mean 6.6, SD 2.3).

There was positive correlation between smartphone skills and student attitude toward improved access to learning material (*r*=.43, *P*<.05), helping to learn more independently (*r*=.44, *P*<.05), and use of smartphones by teaching staff (*r*=.45, *P*<.05) (Table 5).

No significant correlation was found between age, gender, origin, and part-time job, and any of the statements regarding students’ attitude toward smartphones.

**Table 5:** Cross tabulation comparing VAS average score of attitudes toward smartphones (improving access to learning material and courses, helping to learn more independently, and use of smartphones by staff) with level of students’ smartphones skills.

Smartphone skills	Improved access		Used by teaching staff		Independent learning	
	Mean	SD	Mean	SD	Mean	SD
Basic	5.5	2.3	4.7	2.0	4.2	2.1
Intermediate	7.0	1.8	6.2	2.1	5.9	2.1
Advanced	8.1	1.3	7.7	2.0	7.2	1.7

## Discussion

### Principal Findings

The results of the current study demonstrate that most students owned smartphones, were able users, and perceived them as learning tools that allow students to access to learning resources. Resources available without students having to physically visit libraries, desktops, or meeting with colleagues because reliable connectivity to the Internet is ensured by the university wireless connection and 3G services.

On a daily basis at this stage in their education, students are on campus, the last year of training is completed in outplacements often in remote areas. Smartphones have been reported to enable learners and practitioners to access not only learning resources, but also professional advice when used in remote areas [12,26,27], opening opportunities to enhance teaching and learning on the last year of students’ training.

The ubiquitous nature of smartphones is an advantage but it could also be a disruption. This study shows that slightly more than one-half of the students used their smartphones in the lecture theater regularly and often. It is a common debate whether use of connected devices during lectures is a productive activity, as often, such use might be irrelevant to the learning activities.

However, it appears that this phenomenon is here to stay, and it probably reflects the current "multitasking" approach of students to learning. Smartphones are often banned from classes [28], but have the potential to engage students' participation, for instance, by helping students creating their own content.

More than one-half of the students used their smartphones to take pictures of their work, probably preclinical work, since these are their first years of training. Content creation by students opens opportunities for the students to record and share their progress with peers and instructors. Students were inclined to think that smartphones improved access to learning material. However, they were much less positive regarding independence of learning and teaching staff using smartphones. Further exploration would reveal if this attitude might be different in a course with activities facilitated by the usage of smartphones.

The diversity of smartphone operating systems overtime and geographical location makes it necessary to use compatible learning applications. Web-based applications such social media are a good example. In this study 76/204 (37.3%) respondents had an educational application and 126/214 (58.8%) had the university application against 178/201 (88.5%) students using their smartphone to access social media. This finding differs from a study where medical students reported to have multiple educational applications [5]. Perhaps reflecting that there are more medical than dental applications available.

A significant number of those who accessed social media with their smartphones found it of value for learning. Social media blended into traditional educational environments might enhance learning and collaboration despite geographic location [7]. This is promising for courses, as the one in this study, with outplacements activities, and with a portion of students whose vernacular is not the teaching language because social media is shown to improve participation of students whose first language is not English [7].

Social media is more popular among younger students. However, the average general skills with smartphones did not vary significantly among age, gender, or origin. This finding suggests that students out of the millennial group, older than 30, have adapted to the use of smartphones and are skilled users of this technology.

### Future Research

Exploring educators' opinion on the use of smartphones for learning and teaching is not included in the present study. However, many faculties might not feel proficient with this type of technology and might find it disruptive. Using social media as a teaching tool might also require staff to have control over the site content because of the risk of students' inappropriate behaviors, such as breaching patients' privacy and authors' copyrights [8]. Whether students will continue to use social media sites in the same way, if these are moderated or visited by their teachers, is an interesting question to be investigated.

The use of smartphones occurring without teaching staff intervention or guidance is an indication of the educational potential of such devices. Smartphones open opportunities for innovative ways to learn and teach. It is encouraging for instructors searching for new teaching methods to see that learning content is accessible, and interaction is possible through smartphones regardless of teaching staff intervention.

### Conclusions

The results from this study corroborate that students use smartphones and social media for their learning activities even though this technology has not been formally included in the curriculum, and perceive their smartphones as learning tools. This might be an opportunity for teaching staff to use smartphones to enhance students’ learning needs without the constraints of time and location. In light of the results of this study, it appears feasible to develop learning activities involving smartphones. It might be advisable to design learning material that not only allows access through computers but also through smartphones.

## Multimedia Appendix 1

Questionnaire.

## Abbreviations

3G: third generation
ICT: information and communication technology
VAS: visual analogue scale
