# Supplementary material for: Investigating the Use of Smartphones for Learning Purposes by Australian Dental Students
Source: JMIR Mhealth Uhealth. 2014 Apr 30;2(2):e20. doi: 10.2196/mhealth.3120 (PMC4114424; doi:10.2196/mhealth.3120)
Supplement: Supplementary file 1 [file mhealth_v2i2e20_app1.pdf]

Investigating the use of smartphones for learning

Please fill this questionnaire to help us to identify students' uses and attitude towards smartphones for learning. The aim of this questionnaire is to obtain information to design better courses for our students. All data provided will remain confidential. By completing this questionnaire you are indicating your willingness to participate. Your participation is greatly appreciated

Section A

Demographics and Social Characteristics

1. Age: _________

2. Gender: Male ☐ Female ☐

3. Are you an international student Yes ☐ No ☐

4. Do you have a part time job at the side of your studies? Yes ☐ No ☐

   - If yes how many hours do you work per week? _________

Type of smartphone and connection

5. Do you have access to WiFi Internet?
   a. At home Yes ☐ No ☐
   b. At the University Yes ☐ No ☐
   c. Elsewhere Yes ☐ No ☐

6. Do you have a smartphone? Yes ☐ No ☐

   If not, are you planning to buy one in the next 6 months? Yes ☐ No ☐

   If yes:

7. What type of smartphone do you have?
   a. Android based ☐
   b. Iphone ☐
   c. Windows based ☐
   d. Blackberry ☐

8. Does your mobile phone contract allow for Internet data use through the 3G network?
    a. Yes, limited amount of data per month ☐
       - If yes, how many MB per month? …………….
    b. Yes, unlimited amount of data per month ☐
    c. No, I don't have data allowance ☐

9. Do you access the Internet with your smartphone?
    a. Yes, through WiFi connection ONLY ☐
    b. Yes, through WiFi AND my 3G mobile carrier (Telstra, Optus etc) ☐
    c. Yes, through my 3G mobile carrier (Telstra, Optus etc) ONLY ☐
    d. No, I don't use my smartphone for Internet access ☐

10. Which of the following best describes whether you own or are interested in getting a mobile tablet device (ex. Apple iPad, Samsung Galaxy Tab)?
    a. I have a mobile tablet device right now ☐
    b. I am likely to get a mobile tablet device within the next year ☐
    c. I am unlikely to get a mobile tablet device within the next year ☐

General Uses of Smartphones

Section B

11. Can you

a. E- Mail ?	Yes ☐	No ☐
b. Chat with friends?	Yes ☐	No ☐
c. Add comments in social media e.g. Facebook?	Yes ☐	No ☐
d. Listen to music?	Yes ☐	No ☐
e. Manage my schedule?	Yes ☐	No ☐
f. Make bank transactions/shopping?	Yes ☐	No ☐
g. Take pictures?	Yes ☐	No ☐
h. Record movies?	Yes ☐	No ☐
i. Up load pictures on the web e.g. facebook?	Yes ☐	No ☐
j. Up load movies on the web e.g. U tube?	Yes ☐	No ☐
k. Edit pictures?	Yes ☐	No ☐
l. Edit movies?	Yes ☐	No ☐
m. Create and edit texts?	Yes ☐	No ☐
n. Synchronize with your home computer?	Yes ☐	No ☐
o. Create drawings?	Yes ☐	No ☐
p. Create animations?	Yes ☐	No ☐
q. Other ________________	Yes ☐	No ☐

Uses of smart phones for learning

12. Can you

a. Look up your course timetable?	Yes ☐	No ☐
b. Look up L@G* announcements?	Yes ☐	No ☐
c. E mail school staff/classmates?	Yes ☐	No ☐
d. Read lecture notes?	Yes ☐	No ☐
e. Watch lecture capture?	Yes ☐	No ☐
f. Watch instructional movies?	Yes ☐	No ☐
g. Do library /literature searches	Yes ☐	No ☐
h. Surf the web for learning material?	Yes ☐	No ☐
i. Share notes with classmates?	Yes ☐	No ☐
j. Take photos of my work?	Yes ☐	No ☐
k. Make movies of my work?	Yes ☐	No ☐
l. Other ________________	Yes ☐	No ☐

13. Do you have any applications related to Dentistry or Education? Yes ☐ No ☐

14. If yes, which applications are most valuable to your studies?

....

....

15. Do you have the Griffith University smartphone application? Yes ☐ No ☐

If yes, how often do you use it? Regularly ☐ Often ☐ Seldom ☐ Never ☐

*L@G: Learning at Griffith

16. Where do you normally use your smartphone for learning activities?

a.	At the library	Regularly ☐ Often ☐ Seldom ☐ Never ☐
b.	In the lecture theatre	Regularly ☐ Often ☐ Seldom ☐ Never ☐
c.	During Tutorials/ Sim laboratory	Regularly ☐ Often ☐ Seldom ☐ Never ☐
d.	Elsewhere on campus	Regularly ☐ Often ☐ Seldom ☐ Never ☐
e.	On the go e.g. on the bus	Regularly ☐ Often ☐ Seldom ☐ Never ☐
f.	At home	Regularly ☐ Often ☐ Seldom ☐ Never ☐
g.	Other ________________	Regularly ☐ Often ☐ Seldom ☐ Never ☐

17. Do you access social media (facebook, twitter) with your smartphone? Yes ☐ No ☐

18. Have you found any value in social media for your education? Yes ☐ No ☐

19. If yes, how have social media helped you in your studies?

Section C

Student attitudes

Please tell us if you agree or disagree with the statements below by placing a mark on the line.
- A mark right of the middle line indicates **agreement**, a mark left of the middle line indicates **disagreement**.
- The higher your certainty to agree/ disagree the closer your mark to the end of the line. If you are uncertain place the mark in the middle.

Examples:
Dentistry is a very demanding University program

I disagree | I strongly agree

*The red mark in this position indicates **strong agreement** with the statement*

Statements:
1. Smartphones improve access to my courses and learning material

I strongly disagree | I strongly agree

2. Smartphones help me to learn more independently

I strongly disagree | I strongly agree

3. Smartphones should be utilized more by the University/ teaching staff

I strongly disagree | I strongly agree

In which other ways do you think a smartphone can be used for learning purposes?
